# Intussuscepted Metachronous small bowel tumor after treatment for colorectal adenocarcinoma

**DOI:** 10.1016/j.ijscr.2019.05.008

**Published:** 2019-05-10

**Authors:** Yuhamy Curbelo-Peña, David Saavedra-Perez, Tomas Stickar, Joan Molinas-Bruguera, Jordi De Cozar-Duch, Xavier Quer-Vall, Helena Vallverdú-Cartie

**Affiliations:** Department of General and Digestive Surgery, Vic University Hospital, Barcelona, Spain

**Keywords:** Small bowel, Adenocarcinoma, Small bowel resection, Intestinal tumor

## Abstract

**Introduction:**

Small Bowel adenocarcinoma (SBA) is a rare malignant neoplasm without specific signs or symptoms. It’s been associated with late stage disease presentations. Midterm outcomes have suggested that after successful removal of colorectal carcinoma, there is higher risk for developing a further primary (metachronous) colorectal tumor. However when it comes to small bowel, metachronous carcinomas are unusual.

**Case presentation:**

A 46-year-old female who underwent an emergency Hartmann's procedure two years previously and treatment of adjuvant chemotherapy for adenocarcinoma of the sigmoid colon at stage IIB with loco-regional recurrence.

The patient presented with bowel obstruction secondary to a small bowel intussusception, confirmed by computed tomography. An emergent exploratory laparotomy was performed and confirmed of an ileal tumor as the cause of small bowel intussusception and clinical bowel obstruction. Histopathology confirmed a primary small bowel mucinous adenocarcinoma with node metastasis (T3N1M0, stage IIIB).

**Discussion and conclusion:**

Patients who develop a small or large bowel adenocarcinoma have high risk of presenting a second tumor at both sites. Since data available to guide therapeutic decisions for patients presenting with small bowel metachronic tumors are scarce, the role of adjuvant therapy in patients who undergo curative resection remains unclear.

The aim of this manuscript is present a case report of a patient admitted for a rare obstructive small bowel intussusception therefore underwent surgery for metachronic small bowel carcinoma from colorectal primary witch previously treated by surgery and adjuvant chemiotherapy. Studies about strategies for detection at an earlier stage, optimal treatment and prognosis are mandatory for this disease.

## Highlights

• We present a case of a small bowel adenocarcinoma at an advanced stage, following surgery and adjuvant therapy for colorectal adenocarcinoma, with an atypical presentation at the emergency room, as a rare cause of obstructive small bowel intussusception.
• After a successful removal of a carcinoma in the large bowel, there is a higher risk for developing a further primary (metachronous) large bowel tumor. However metachronous carcinomas affecting small bowel are rarer.
• Small Bowel adenocarcinoma (SBA) is a rare malignant neoplasm without specific signs and symptoms, and associated with late stage disease presentations.
• Patients who develop a small or large bowel adenocarcinoma are at high risk for a second cancer at both sites. However data available to guide therapeutic decisions for those presenting one at small bowel are scarce, and the role of adjuvant therapy in patients who undergo curative resection is unclear.
• Studies about strategies for detection at an earlier stage, optimal treatment and prognosis are mandatory for this disease.

## 1 Introduction

Five percent of malignant neoplasms of the gastrointestinal (GI) tract occur in the small bowel [1]. Adenocarcinoma is the most common histopathology with the following distribution: 56% duodenum, 16% jejunum, 13% ileum, and 15% not identified [2]. The annual incidence of small bowel adenocarcinoma (SBA) in the USA is approximately 3.9 cases per million persons with age ranging between 60 and 70 years [3]. Most of them are asymptomatic until complications such as bleeding, perforation or intestinal obstruction have appeared. Nonspecific signs and symptoms associated with difficulty in performing small bowel examination is the cause of a delayed diagnosis and typically late stage presentations [3]. Small bowel intussusception is a common condition in childhood, mostly for an idiopathic cause. However, it is possible to see this pathology exceptionally in adults, with a 90% of cases due to an organic disease [3].

After successful colorectal cancer removal, there is high risk of developing a further primary (metachronous) large bowel tumor [3]. However, metachronous carcinomas affecting the small bowel are rarer. We present a case of a small bowel adenocarcinoma at an advanced stage, following surgery and adjuvant chemotherapy for colorectal adenocarcinoma, with atypical presentation at emergency room, as a rare obstructive small bowel intussusception. This case is reported in line with the SCARE criteria and PROCESS guidelines [4].

## 2 Case report

46-year-old female with past surgical history of Hartmann’s procedure on 2017 for abscessed and obstructive adenocarcinoma of the sigmoid. Histopathological study confirmed low-grade mucinous adenocarcinoma of sigmoid colon with involvement of all layers and perforation of the visceral peritoneum, pT4pN0. K-RAS gene mutation was present thus chemotherapy with capecitabine was completed. Hartmanns’s reversal procedure was performed months later.

Thereafter 18-months-postoperative follow up appointment, computed tomography (CT) revealed iliac lymphatic recurrence. FOLFOX and FOLFIRI-aflibercept chemotherapy were provided. Abdominal CT control confirmed the persistence of lymphatic disease and target sign in small bowel suggesting intussusception as well.

**Fig. 1:**
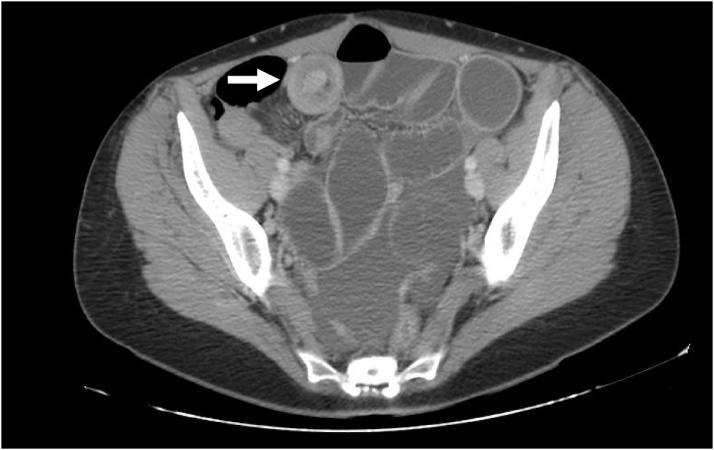
Abdominal CT evidenced a complete bowel obstruction secondary to intestinal caliber change, at the level of bowel intussusception (arrow showing the ´doughnut sign´).

**Fig. 2:**
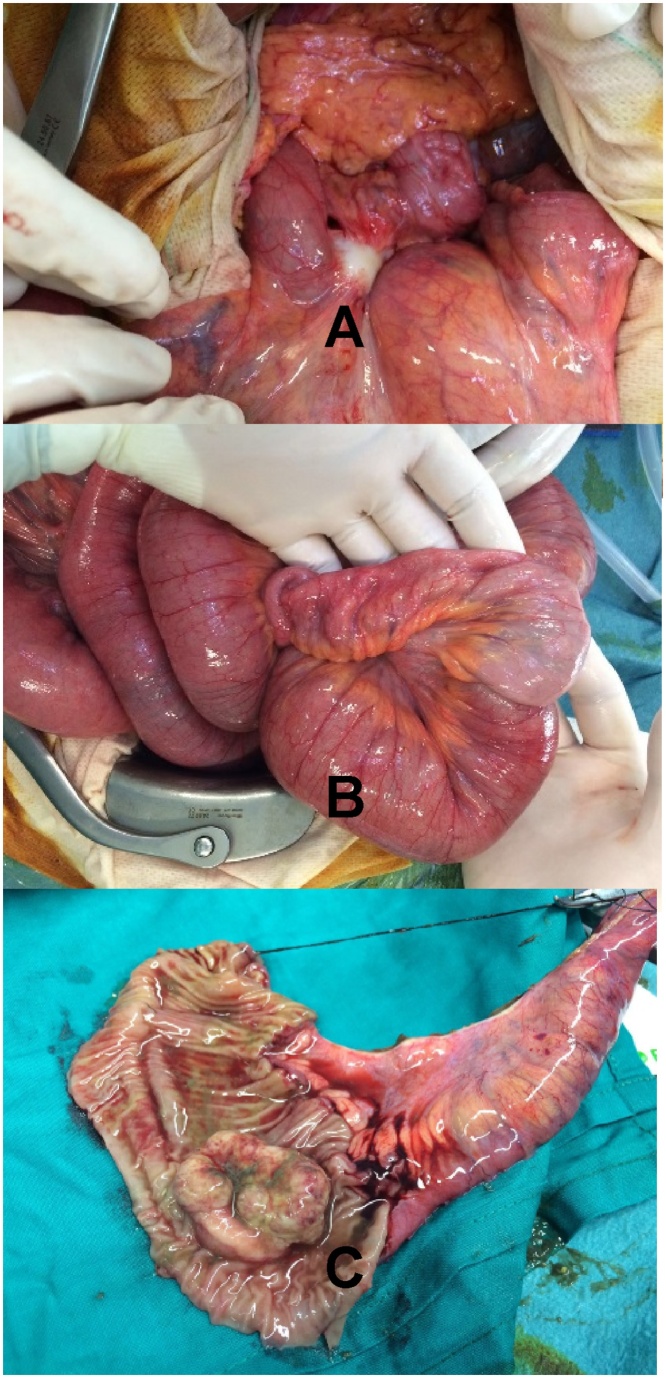
Intraoperative findings: A: Local recurrence in left iliac chain. B: Ileum-ileal intussusception at 50 cm from the ileocecal valve. C: Small bowel adenocarcinoma.

Forty-eight hours later, the patient presented to emergency department complaining of abdominal pain and distension, lack of elimination of flatulence and vomiting. An abdominal X-ray showed dilated bowel loops with air-fluid levels. Blood test revealed normal white cells level, and a serum-C-reactive protein of 206 mg/L. Abdominal CT evidenced a complete bowel obstruction secondary to small bowel intussusception (Fig. 1). The laparotomy confirmed small bowel obstruction, dependent on intussusception at 50 cm from the ileocecal valve (Fig. 2: intraoperative findings) and the lymphatic recurrence as well. Small bowel resection with mechanical side to side anastomosis were performed. The histopathological analysis confirmed primary small bowel mucinous adenocarcinoma with lymph node metastasis (stage IIIB, T3N1M0). Consecutive both radiotheraphy and chemotherapy with FOLFOX were concluded.

After eight months on follow up, the patient had an elevation of tumor markers level. Abdominal CT showed left iliac lymph node disease, and PET Scan settled extra focus at retroperitoneum. The patient is about to start additional chemotherapy treatment.

## 3 Discussion

Since SBA are exceptional tumors, the current evidence available remains hesitant. Therefore the specific clinical characteristics, the best treatment modalities and prognosis are not clear [3].

Most of the adenomas in the small bowel have origin on duodenum, with malignancy incidence reported of 42%. Villous histopathology, growing size and higher grade of dysplasia increase the risk of malignancy [3]. Some authors have proposed that following colorectal cancer the risk of SBA was elevated. On the other hand, a 4–5-fold risk of colorectal cancer following primary SBA, has been described as well [1,3]. These outcomes suggest etiological similarities between these tumors, nonetheless potential common carcinogenic agents have not been reported so far [3].

On clinical presentation, the most common symptoms of SBA are: abdominal pain, weight loss, nausea, vomiting, and gastrointestinal bleeding. Small bowel Intussusception is exceptionally in adults, with 90% of cases secondary to organic disease. Abdominal CT findings such as ‘target’ or ‘doughnut’ sign, may indicate the presence of small bowel intussusception, as previous case.

SBA diagnosis can be assessed by enteroclysis in 90% of cases, whereas only 33% of them could be identified with barium follow-through study [1,3]. The overall sensitivity, specificity, and accuracy of multidetector row helical computed tomography (CT) enteroclysis for small bowel disease are 100%, 95%, and 97%, respectively, and 86%, 98%, and 97%, respectively, for magnetic resonance (MR) enteroclysis. Video capsule enteroscopy (VCE) was introduced in 2001 and its diagnostic yield is approximately 50–60% for small bowel lesions [2,3]. Despite that, there is an average delay in diagnosis reported of 8.2 months attributable to physician failure to order the appropriate tests and 12 months-delay due to the radiologist failure to confirmed diagnosis, as in our case [3]. On diagnosis and follow-up, the value between tumor markers and SBA is controversial. Some patients with SBA have been found elevated their serum CA19-9 or CEA concentrations but is not the rule [3]. We did not found this in our patient.

SBA staging is performed according to the American Joint Committee on Cancer (AJCC) guidelines, which is based on the TNM staging system [3]. The mainstay treatment of SBA remains surgical resection of the involved segment, the mesentery and the lymphatics up to the superior mesenteric vessels [3]. Morbidity is around 13% and 44% and mortality between 3% and 12%. Specific survival by stage was 65% for stage I, 48% for stage II, 35% for stage III, and 4% for stage IV [2,3], and the 5-year overall survival varies between 9% and 50% [3]. Treatment with capecitabine or infusional 5-FU combined with oxaliplatin appears to be one of the most active combinations and should be considered for front line treatment (median survival between 14–20 months) [3]. Nevertheless, the role and benefit of adjuvant chemotherapy still unclear, just few single-institution retrospective studies have evaluated these chemotherapy modalities, as in our patient who presented metachronous primary SBA despite completing adjuvant chemotherapy. More studies are mandatory to improve outcomes.

Several factors have been associated with poor prognosis, the most important are late staging, lymph node involvement, poor histopathology differentiation, old age, duodenal primary, and positive margins [2,3]. However, it was described by Howe et al, according to the National Cancer Database (NCDB), that the only factor related to Overall Survival of SBA is the patient age, since relative risk of death was 1.8 times higher for patients older than 75 years. Recurrences were observed in 40–70% of patients who underwent curative resection, with most recurrences at distant sites [3]. Early diagnostic and complete surgical resection remains the most significant variables in improving the outcome of patients with SBA. However, more studies will be needed to clarify this issue.

## 4 Conclusion

Metachronous carcinomas affecting both large or small bowel are a very unusual condition. Poor prognosis is related to elderly patients in contrast to our young female patient, who despite her young age, developed colon carcinoma and later on, another primary SBA. We highlight the patient's inadequate response to chemotherapy treatment with a second gastrointestinal carcinoma and evolution to intussusception and finally obstruction, besides treatment. We consider our case an exceptional presentation that represents a challenge for any surgeon on diagnosis, treatment and follow-up of patients with GI adenocarcinoma.

## Conflict of interest

We declare no conflicts of interest.

## Sources of funding

We have no any source of funding to declare.

## Ethical approval

We obtained the patient’s informed consent besides our case report is exempt from ethnical approval in our institution.

## Consent

The authors have obtained the written and signed consent to publish the case.

## Author contribution

Author 1 GARANTOR - patient care, study concept or design, data collection, data analysis or interpretation, writing the paper, Images and revision.

Author 2- patient care, study concept or design, data collection, data analysis, writing the paper, Images and revision.

Author 3- patient care, study concept or design, data collection, data analysis, writing the paper, Images and revision.

Author 4- patient care, study concept or design, data collection, data analysis, writing the paper, Images and revision.

Author 5- patient care, study concept or design, data collection, data analysis, writing the paper, Images and revision.

Author 6- patient care, study concept or design, data collection, data analysis, writing the paper, Images and revision.

Author 7- patient care, study concept or design, data collection, data analysis, writing the paper, Images and revision.

## Registration of research studies

N/A.

## Guarantor

Yuhamy Curbelo-Peña, MD.

## Provenance and peer review

Not commissioned, externally peer-reviewed.
